# The effect of Hydrocortisone on implantation through upregulation of tight junction genes: A lab trial study

**DOI:** 10.5935/1518-0557.20250027

**Published:** 2025

**Authors:** Sahar Eghbali, Hossein Eyni, Aryan Ayati, Mehrdad Ghorbanlou, Marzieh Ajdary, Fatemehsadat Amjadi, Mehdi Mehdizadeh, Fatemeh Moradi, Mehrdad Bakhtiyari

**Affiliations:** 1 Department of Anatomy, Iran University of Medical Sciences, Tehran, Iran; 2 Student Research Committee, Iran University of Medical Sciences, Tehran, Iran; 3 Department of Anatomy, School of Medicine, Iran University of Medical Sciences, Tehran, Iran; 4 Stem Cell and Regenerative Medicine Research Center, Iran University of Medical Sciences, Tehran, Iran; 5 Research Center for Advanced Technologies in Cardiovascular Medicine, Cardiovascular Diseases Research Institute, Tehran University of Medical Sciences, Tehran, Iran; 6 Endometriosis Research Center, Iran University of Medical Sciences, Tehran, Iran; 7 Reproductive Sciences and Technology Research Center, Department of Anatomy, Iran University of Medical Sciences, Tehran, Iran; 8 Cellular and Molecular Research Center, Iran University of Medical Sciences, Tehran, Iran

**Keywords:** hydrocortisone, tight junctions, endometrium, implantation

## Abstract

**Objective:**

Emotional stress leading to cortisol release is an important infertility factor in females. Increased cortisol levels can significantly affect tight junction proteins and dysregulate the implantation process. This study investigated the effect of exogenous synthetic Cortisol (Hydrocortisone) on the expression of specific genes encoding several junctional proteins in the endometrial epithelial cells.

**Methods:**

Following endometrium sampling from 25 participants, the biopsied tissue was digested and filtered through a cell strainer to prepare endometrial epithelial cells. After confluency, cells were treated with 50, 100, and 200 nM hydrocortisone concentrations and incubated for 24, 48, and 72 hr with repeated treatments every 24hr. qPCR analysis of 4 tight junction genes, including CLDN3, CLDN4, ZO-1, DSG1, and CDH1 was performed. Gene expressions were reported using a relative quantification method.

**Results:**

Higher tight junction gene expression was evident at higher concentrations of Hydrocortisone. A significant increase in expression of CLDN3, CLDN4, ZO-1, DSG1 and CDH1 was observed at 100 nm concentrations of Hydrocortisone compared with the control group during different treatment durations.

**Conclusions:**

In conclusion, Hydrocortisone treatment (at 100 nm concentration) significantly increased the expression of tight junction genes, suggesting that the blastocyst cannot infiltrate the endometrium readily, thus preventing implantation. Glucocorticoids can disrupt implantation by influencing tight junction molecule expression. Thus, the physicians must further investigate the effect of glucocorticoids treatments on implantation

## INTRODUCTION

Although the prevalence of infertility is not clearly known, it is estimated that female infertility affects 15% of all women worldwide (Nik Hazlina *et al*., 2022), and 37% of infertile couples struggle with female infertility (Maharlouei *et al*., 2021). Several leading causes have been reported for female infertility, including ovarian factors (e.g., polycystic ovary syndrome), body weight (either underweight or overweight), emotional stress, tubal and peritoneal factors (e.g., inflammatory disease), endometriosis, Fallopian tube obstruction, anomalies (uterine, tubule, ovarian or cervical anomalies), advanced ages (>35 yr), hormonal disorders, smoking, genetic factors (Mustafa *et al*., 2019).

Among the causes of female infertility, emotional stress is an essential factor that has been discussed in previous studies in this study. Stress influences the hypothalamic-pituitary-adrenal axis, leading to impairment of gonadotropin-releasing hormone, prolactin, luteinizing hormone, and follicular stimulating hormone (Direkvand-Moghadam *et al*., 2013; Rooney & Domar, 2018). As the primary glucocorticoid secreted in the adrenal cortex’s zona fasciculate, Cortisol is affected by factors associated with the hypothalamic-pituitary-adrenal axis (García-León *et al*., 2019). Several studies have introduced Cortisol as the “stress hormone” and its adverse effect on female fertility (Alam *et al*., 2020; More *et al*., 2022; Wdowiak *et al*., 2020).

As one of the most critical stages of pregnancy, implantation is susceptible to stress (Deaton *et al*., 2018). The blastocyst must penetrate the endometrium for implantation to occur. The epithelial cells of the endometrium, like other epithelial cells, contain desmosomal proteins Desmoglein-1 (DSG1), adherens junction proteins Cadherin-1 (CDH1), and tight junction proteins (Zonula Occludens-1 (ZO-1), and claudin-4 (CLDN4)) (Ye, 2020). There are alterations in the expression of these proteins at specific sites of the endometrium known as the implantation window. These alterations contribute to blastocyst implantation (Grund & Grümmer, 2018). In some studies, Cortisol has been reported to increase Claudin-4, occludin, and E-cadherin expression in junctional proteins (Huang *et al*., 2020; Welcome & Mastorakis, 2020).

This study aimed to the effect of exogenous synthetic Cortisol (Hydrocortisone) on the expression of specific genes encoding several junctional proteins in the endometrial epithelial cells. To assess this interaction, the expression of specific genes encoding junctional proteins in the endometrial epithelial cells was analyzed with and without exposure to Cortisol as a marker for stress.

## MATERIALS AND METHODS

### Reagents

After confluence, cells were treated with 50, 100, and 200 nM hydrocortisone levels and incubated for 24, 48, and 72 hr with repeated treatments every 24 hr. Quantitative polymerase chain reaction (qPCR) analysis of 5 tight junction genes, including Claudin-3 (CLDN3), CLDN4, ZO-1, DSG1, and CDH1 was performed. Gene expressions were reported using a relative quantification method.

The culture medium, Dulbecco’s Modified Eagle Medium (DMEM/F12) (Gibco, Scotland, UK), fetal bovine serum (FBS) and L-Glutamine (Invitrogen, Grand Island, US), penicillin/streptomycin (Sigma-Aldrich, Steinheim, Germany) were used for cell culture, and Hydrocortisone (Sigma-Aldrich, Tauf- Kirchen, Germany) was used for cell treatment. TAKARA PCR Thermal Cycler Dice (TakaRa, Otsu, Japan), Trizol reagent (Sigma-Aldrich, Pool, UK), Stratagene Mx3005 P, Strata Script First-Strand Synthesis System and Velocity-SYBR Green QPCR master mix (STRATA-GENE Company, Cedar Greek, TX, USA) were used for quantitative real-time PCR.

### Patient and Grouping

Endometrial sampling was performed in women meeting the inclusion criteria to extract human endometrial cells. 25-35 yr-old women referred to the infertility clinic due to male factor infertility were included in the study at about the 19th-23rd day of their menstrual cycle. An experienced gynecologist extracted all the samples under aseptic conditions. Informed consent was obtained from all participants. Due to ethical issues and limitation in human sample, this sample size was chosen.

### Cell culture

The biopsied endometrium was washed in Phosphate-buffered saline (PBS) containing penicillin/streptomycin three times (Huang *et al*., 2020; Lee *et al*., 2020). Subsequently, the tissue was cut into 2-3 mm pieces and incubated in a digesting solution containing 1mg/ml collagenase type 1A, DMEM, and 10% FBS for 45 minutes. The resulting suspension was treated with red blood cell lysis buffer (ammonium chloride), potassium bicarbonate (Sigma-Aldrich, Steinheim, Germany), and Ethylenediaminetetraacetic acid (EDTA) (500 mM, Teknova, California, USA)) for 5 min. Afterward, it was filtered through a 40 and 70 µm cell strainer to separate the suspension into stromal and epithelial compartments, respectively. The suspension containing epithelial cells was centrifuged at 1300 Revolutions Per Minute (RPM) for 5 min, then 5 ml of DMEM-F12, FBS 5%, L-Glutamine 1%, and penicillin/streptomycin 1% was added to the cell pellet and incubated in a 25ml culture flask at 95% humidity, 5% Co2, and 37oC. Following confluency, cells were treated with 50, 100, and 200 nM hydrocortisone and incubated for 24, 48, and 72 hr while repeating the treatment every 24 hr.

### Real-time polymerase chain reaction analysis

Following the manufacturer’s instructions, Trizol reagent was utilized to extract the total Ribonucleic acid (RNA) (Zong *et al*., 2019). The TAKARA PCR Thermal Cycler Dice was employed for PCR amplification. For synthesizing complementary Deoxyribonucleic acid (DNA) (cDNA), the Strata Script First-Strand Synthesis System with an oligo-dT primer was employed, using 50 ng of total single-stranded RNA template. To perform quantitative real-time PCR (RT-PCR) analysis, the Stratagene Mx3005 P instrument with Full Velocity-SYBR Green QPCR master mix was utilized, following the manufacturer’s protocols (Yu *et al*., 2020). The primer sequences used in the study can be found in Table 1. The human β-actin gene served as an internal control for normalization. The β-actin is commonly used as a housekeeping gene for RT-PCR and Western Blot because it is regarded as a highly stable housekeeping gene (Ruan & Lai, 2007). The amplification process followed a specific temperature profile: an initial step at 95°C for 10 min, followed by cycles of denaturation at 95°C for 15 sec, and annealing/extension at 60°C for 1 min, repeated for 40 cycles. Each sample was tested in triplicate. A melting curve analysis was performed to determine the specificity of the PCR fragments, resulting in a single peak for each PCR product. Standard curves were generated by performing logarithmic serial dilutions of total cDNA. The cycle threshold (CT) values were normalized against the CT value of the human β-actin gene. The comparative CT method was employed to analyze the qPCR data.

**Table 1:** Primer sequences and traits.

Gene	FS	RS	AT	PS
CLDN3	5'TGACCGCCAATACTGACCA3'	5'AATATCAAGTGCCCCTAGG3'	60	101
CLDN4	5'CTCTGCGAACGTTACAGG3'	5'TGCCCATTACCTGTAGCCC3'	60	101
ZO-1	5'TACCAGTAAGTCGTCCTGATCC3'	5'ACTCCTTCTGTTAACCACACCA3'	60	113
DSG1	5'TCCGAAGGCAGAATGAAT3'	5'TTTGGCGATTGGGTTCCTCT3'	60	86
CDH1	5'TGCTCGTGTTTGACTAAGG3'	5'TGGTCTTTGTCTGACTCTG3'	60	81
β-actin	5'TTCTAACCCGTGACTATGCATA3'	5'AATCTATGGGGTGACTATGCAAAT3'	60	103

FS: Forward Sequence, RS: Reverse Sequence, AT: Annealing Temperature, PS: Product Size.

In essence, the difference in cycle time (DCT) represents the disparity between the number of cycles required for amplifying the target gene and the reference gene, which in this case was human β-actin. The DDCT value was determined by calculating the difference between the experimental groups. The fold-change (FC) was computed using the formula FC = 2^DDCT.

### Statistical analysis

Data were presented as means ± standard error of the mean from triplicates. One Way Analysis of Variance (ANOVA, post hoc Tukey) was used for comparing the difference of normally distributed means in multiple tests. For real-time PCR analysis, the relative quantification method was applied. Data were analyzed by SPSS 16 software (SPSS, Inc., Chicago, IL, USA), and *p* values of ≤0.05 were considered significant.

## RESULTS

### CLDN3 gene expression

**Figure 1:**
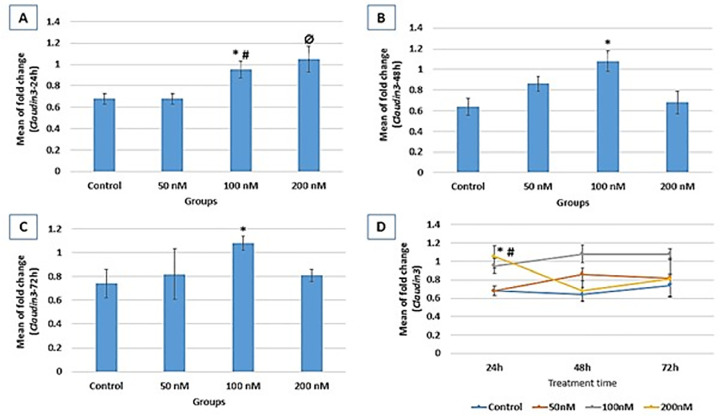
Relative expression fold change of CLDN3 at 3 different treatment times (A: 24 hr, B: 48 hr, and C: 72 hr). Gene expression results are normalized to β-actin mRNA amplification. A: * *vs*. control (*p*=0.003); # *vs*. 50nM (*p*=0.014); Ø *vs*. control and 50nM (*p*=0.002); B: * *vs*. control and 200nM (*p*=0.002); C: * *vs*. control (*p*=0.04); D: CLDN3 expression process within each group through study course; 200nM (* *vs*. 48h (*p*=0.009), # *vs*. 72h (*p*=0.05).

Following 24h treatment of endometrial epithelial cells with Hydrocortisone, CLDN3 expression was significantly higher in the100 nM (0.95±0.08) and 200 nM (1.05±0.12) group compared to the control group (0.68±0.05; *p*=0.01, and 0.002, respectively). After 48h of Hydrocortisone treatment, CLDN3 expression was significantly increased in 100 nM group (1.08±0.1) compared to the control (0.64±0.08) group (*p*=0.002). Finally, following 72h of treatment, CLDN3 expression was increased significantly in the 100nM group (1.08±0.06) compared to the control group (0.74±0.12, *p*=0.04). Through the study course, CLDN3 expression was evaluated at three-time points of treatment. Alterations of CLDN3 expression at these time points in each group were not significant, except for the 200nM group showing significantly higher expression at 24 hr compared to 48 hr (*p*=0.009) and 72 hr (*p*=0.05) treatments (Figure 1).

### CLDN4 gene expression

**Figure 2:**
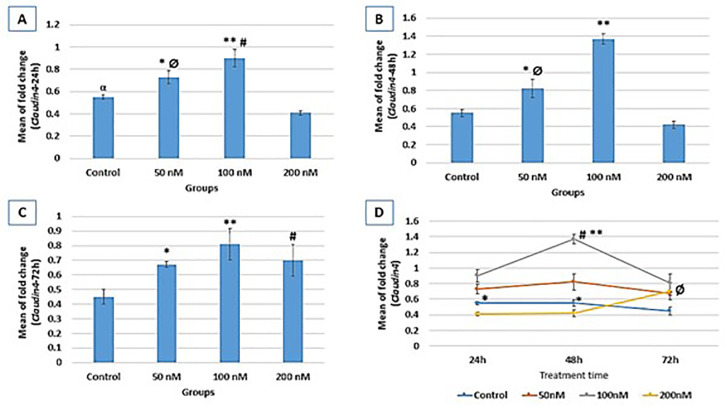
Relative expression fold change of CLDN4 at three different treatment times (A: 24h, B: 48h, and C: 72h). Gene expression results are normalized to β-actin mRNA amplification. A: **vs. control and 200nM (*p*=0.000); # *vs*. 50nM (*p*=0.01); * *vs*. control (*p*=0.01); Ø *vs*. 200nM (*p*=0.000); α *vs*. 200nM (*p*=0.03); B: ** *vs* control, 50nM, and 200nM (*p*=0.000); * *vs*. control (*p*=0.004); Ø *vs*. 200nM (*p*=0.000); C: ** *v*s. control (*p*=0.003); # *vs*. control (*p*=0.02); * *vs*. control (*p*=0.04); D: CLDN4 expression process within each group through study course; control (* *vs*. 72h (*p*=0.04); 100nM (# *vs*. 24h (*p*=0.001), ** *vs*. 72h (*p*=0.000)); 200nM (Ø *vs*. 24h and 48h (*p*=0.005)).

CLDN4 expression following 24h treatment exhibited a significant increase in 100nM and 50nM groups (0.9±0.08) compared to the control (0.55±0.02) group (*p*=0.01). CLDN4 expression was also significantly lower in the 200nM compared to the control (*p*=0.03). Following 48h of treatment, CLDN4 expression was significantly increased in the 100 nM (1.37±0.06) and 50nM group compared to the control (0.55±0.04, *p*=0.000and 0.004, respectively) (Figure 2). After 72 hr of treatment, a significant increase in CLDN4 expression was evident in 100 nM (0.81±0.11), 200 nM (0.7±0.11), and 50 nM (0.67±0.02) groups compared to the control group (0.45±0.05) with *p*-values of 0.003, 0.02 and 0.04, respectively.

Alterations of CLDN4 expression at three-time treatment points in each group revealed significantly higher expression at 24 hr and 48 hr compared to 72 hr treatment (*p*=0.04) in the control group. In the 100 nM group, significantly higher expression was evident at 48 hr compared to 24 hr (*p*=0.001) and 72 hr (*p*=0.000) of treatment. In the 200 nM group, a higher expression was detected at 72 hr compared to 24 hr and 48 hr (*p*=0.005) treatment (Figure 2).

### ZO-1 gene expression

**Figure 3:**
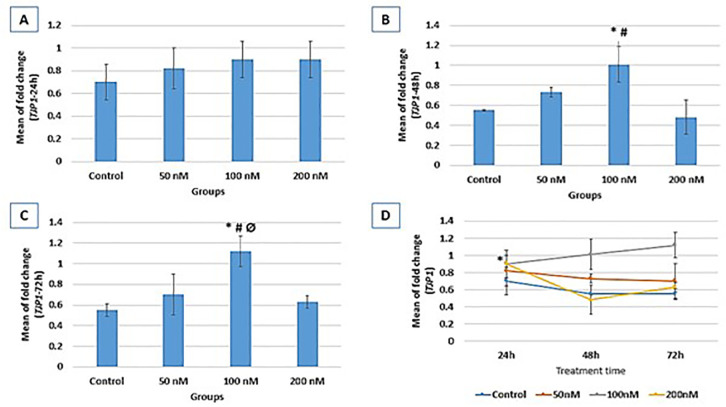
Relative expression fold change of ZO-1 at three different treatment times (A: 24h, B: 48h, and C: 72h). Gene expression results are normalized to β-actin mRNA amplification. B: * *vs*. control (*p*=0.009); # *vs*. 200nM (*p*=0.004); C: * *vs*. control (*p*=0.003); # *vs*. 50nM (*p*=0.019); Ø *vs*. 200 (*p*=0.008); D: ZO-1 expression process within each group through study course; 200nM (* *vs*. 48h (*p*=0.02)).

According to figure 3, ZO-1 expression following 24 hr of treatment indicated no significant difference between the four groups. Following 48 hr of treatment, ZO-1 expression was significantly increased in the 100 nM group (1.01±0.18) compared to the control (0.55±0.01, *p*=0.009). After 72 hr of treatment, ZO-1 expression was also significantly higher in the 100 nM group (1.12±0.15) compared to the control (0.55±0.06, *p*=0.003). ZO-1 expression showed no significant change through the study course except for the 200 nM group exhibiting significantly higher expression at 24 hr compared to 48 hr and 72 hr (*p*=0.02) treatments (Figure 3).

### CDH1 gene expression

**Figure 4:**
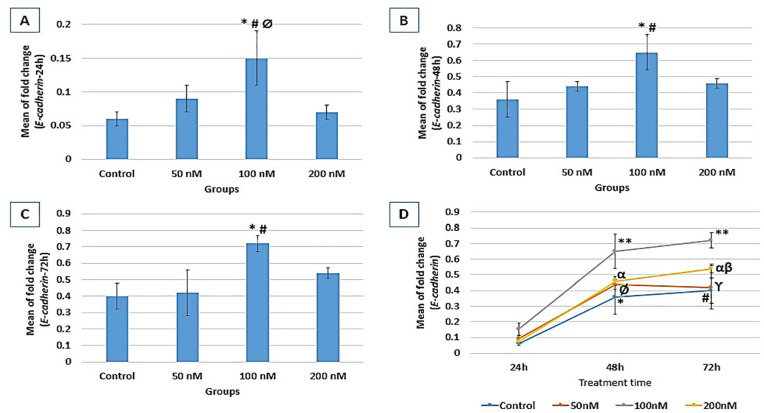
Relative expression fold change of CDH1 at three different treatment times (A: 24h, B: 48h, and C: 72h). Gene expression results are normalized to β-actin mRNA amplification. A: * *vs*. control (*p*=0.007); # *vs*. 50nM (*p*=0.05); Ø *vs*. 200nM (*p*=0.01); B: * *vs*. control (*p*=0.01); # *vs*. 50nM (*p*=0.05); C: * *vs*. control (*p*=0.008); # *vs*. 50nM (*p*=0.01); D: CDH1 expression process within each group through study course; control (* *vs*. 24h (*p*=0.008), # *vs*. 24h (*p*=0.004)); 50nM (Ø *vs*. 24h (*p*=0.005), ϒ *vs*. 24h (*p=*0.007)); 100nM (** *vs*. 24h (*p*=0.000)); 200nM (α *vs*. 24h (*p*=0.000), β *vs*. 48h (p=0.01)).

CDH1 expression was significantly higher in the 100 nM group compared to the control in all three-time points of 24 hr (*p*=0.007), 48 hr (*p*=0.01), and 72 hr (*p*=0.05). CDH1 expression increased with time, revealing higher expressions at 48 hr and 72 hr compared to 24 hr of treatment. In the control group, significantly higher expressions were indicated at 48 hr and 72 hr compared to 24 hr (*p*=0.008 and *p*=0.004, respectively) treatment (Figure 4). In the 50 nM group, a significant increase in CDH1 expression was evident at 48 hr and 72 hr compared to 24 hr (*p*=0.005, and 0.007, respectively) treatment. In 100nM and 200nM groups, there was also a significantly higher expression at 48 hr and 72 hr compared to 24 hr (*p*=0.000) treatment. In the 200 nM group, CDH1 expression at 72 hr was significantly higher than the 48 hr (*p*=0.01) treatment.

### DSG1 gene expression

**Figure 5:**
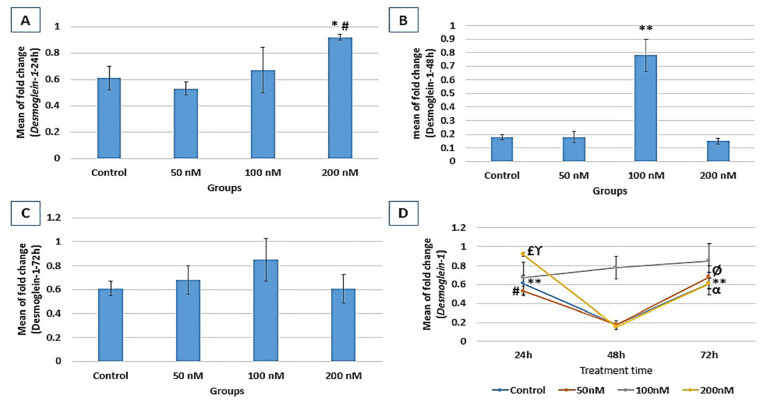
Relative expression fold change of DSG1 at three different treatment times (A: 24h, B: 48h, and C: 72h). Gene expression results are normalized to β-actin mRNA amplification. A: * *vs*. control (*p*=0.02); # *vs*. 50nM (*p*=0.006); B: ** *vs*. control, 50nM, and 100nM (*p*=0.000). D: DSG1 expression process within each group through the study course; control (** *vs*. 48h (*p*=0.000)); 50nM (# *vs*. 48h (*p*=0.004), Ø *vs*. 48h (*p=*0.001)); 200nM (£ *vs*. 48h (*p*=0.000), ϒ *vs*. 72h (*p*=0.004), α *vs*. 48h (*p*=0.001)).

According to figure 5, DSG1 expression following 24h treatment showed a significant increase in the 200nM group (0.92±0.02) compared to the control (0.61±0.09, *p*=0.02). Following 48h treatment, DSG1 expression significantly increased in the 100 nM group (0.78±0.12) compared to the control (0.18±0.02) group (*p*=0.000). After 72 hr treatment, although DSG1 expression in all the groups was higher than 48 hr treatment, no significant difference was evident between groups. In the control group, DSG1 expression was significantly higher at 24 hr and 72 hr compared to 48 hr (*p*=0.000) treatment. In the 50 nM group, there was a significantly higher expression at 24 hr and 72 hr compared to 48 hr (*p*=0.004, and *p*=0.001, respectively) treatment. In the 200 nM group, there was a significant increase in DSG1 expression at 24 hr and 72 hr compared to 48 hr (*p*=0.000, and *p*=0.001, respectively) treatment. There was also a significantly higher expression at 24 hr compared to 72 hr (*p*=0.004) treatment in the control group (Figure 5).

## DISCUSSION

According to the results, Hydrocortisone increases the expression of genes associated with tight junctions in the human endometrial epithelium. As more tight junctions occur between epithelial cells in the endometrium, the more difficult it is for blastocysts to penetrate the endometrium, compromising implantation.

Tight junction molecules are presented on epithelial cells’ lateral and basolateral surfaces. As reported in several in vitro studies, glucocorticoids increase the expression of their receptors upon entering the cytoplasm. The increased receptors result in a greater concentration of ligand-receptor complexes acting as direct and indirect transcription factors, promoting gene expression associated with tight junctions (Lee *et al*., 2020; Zong *et al*., 2019). This increase in the tight junctions makes the epithelium less penetrable, improving the function of the epithelium as a barrier and an integrated structure. The formation of an alveolocapillary barrier in human lung epithelial cell lines was also reported following glucocorticoid treatment and its effect on CDH1 expression (Atherly *et al*., 2019; Yu *et al*., 2020).

In this study, higher cortisol concentration was directly associated with the upregulation of tight junction proteins, reaching its maximum at 100nm cortisol levels. However, Hydrocortisone’s tight junction-associated gene expression is decreased in 200nm concentrations. There are two isoforms of glucocorticoid receptors, glucocorticoid receptor (GRα) and glucocorticoid receptor (GRβ) (Hutter *et al*., 2019; Ramos-Ramírez & Tliba, 2021). GRα binds to DNA and other transcription factors, altering specific gene regulations. In contrast, GRβ binds to DNA and forms a homodimer, preventing the binding of GRα and inhibiting transcription. Thus, the balance between these two receptors determines the sensitivity of cells to glucocorticoids. An increased amount of GRβ in the cells leads to glucocorticoid resistance. Therefore, it is probable that high ligand concentrations and GRβ receptors’ saturation result in decreased gene expression (Ramos-Ramírez & Tliba, 2021). Our study also revealed decreased gene expression at a high Hydrocortisone (200 nm) concentration. Glucocorticoid-derived downregulation of glucocorticoid receptors was reported in another study in which a 100nM concentration of dexamethasone was reported as the most efficient dose of this glucocorticoid (Díez-Tercero *et al*., 2022).

Glucocorticoids can have a double-edged sword role on implantation since they may adversely affect implantation by upregulating tight junctions. Conversely, they may suppress immunologic rejection of embryos during implantation by reducing natural killer cells (Buck *et al*., 2012). This study revealed that the most efficient dosage and treatment duration with Hydrocortisone was 100 nm in 48 hours, whereas, in 200 nm hydrocortisone, the expression of junctional molecule genes decreased after 72 hours. These results indicate that higher concentrations for a prolonged duration may conversely affect tight-junction genes. Studies have reported a higher amount of NK cells in women experiencing recurrent abortions, and when these women were treated with methylprednisolone (which suppresses NK cells), conception rates were increased (Andreescu *et al*., 2023; Fu *et al*., 2021). It has also been reported that NK cells are responsible for glucocorticoid receptor expression (Capellino *et al*., 2020; Quatrini *et al*., 2021). Another recent study (Zhaeentan *et al*., 2018) showed that upon treatment of fallopian tube epithelial cells with Hydrocortisone, tight junction molecule expression was significantly increased, leading to a lower chance of implantation, which is essential in reducing ectopic implantation in the fallopian tube.

## CONCLUSION

Glucocorticoids (Hydrocortisone) influence gene expression associated with tight junction molecules in a concentration and time-dependent manner. It was revealed that the highest tight junction molecule expression was at 100nm for 48hr of treatment. The conflicting role of glucocorticoids on implantation must be considered when specialists prescribe them.

## Abbreviations

qPCR: Quantitative polymerase chain reaction, CLDN3: Claudin-3, CLDN4: Claudin-4, ZO-1: Zonula Occludens-1, DSG1: Desmoglein-1, CDH1: Cadherin-1, DMEM: Dulbecco’s Modified Eagle Medium, FBS: Fetal bovine serum, PBS: Phosphate-buffered saline, EDTA: Ethylenediaminetetraacetic acid, RPM: Revolutions Per Minute, CT: Cycle Threshold, DCT: Difference Cycle Time, FC: Fold-Change, FS: Forward Sequence, RS: Reverse Sequence, AT: Annealing Temperature, PS: Product Size.
